# Impact of education and provision of complementary feeding on growth and morbidity in children less than 2 years of age in developing countries: a systematic review

**DOI:** 10.1186/1471-2458-13-S3-S13

**Published:** 2013-09-17

**Authors:** Zohra S Lassi, Jai K Das, Guleshehwar Zahid, Aamer Imdad, Zulfiqar A Bhutta

**Affiliations:** 1 Division of Women and Child Health, The Aga Khan University, Karachi, Pakistan; 2 Global Child Health and Policy, Centre for Global Child Health, The Hospital for Sick Children, Toronto, ON, Canada

## Abstract

**Background:**

About one third of deaths in children less than 5 years of age are due to underlying undernutrition. According to an estimate, 19.4% of children <5 years of age in developing countries were underweight (weight-for-age Z score <-2) and about 29.9% were stunted in the year 2011 (height-for-age Z score <-2). It is well recognized that the period of 6-24 months of age is one of the most critical time for the growth of the infant.

**Methods:**

We included randomized, non-randomized trials and programs on the effect of complementary feeding (CF) (fortified or unfortified, but not micronutrients alone) and education on CF on children less than 2 years of age in low and middle income countries (LMIC). Studies that delivered intervention for at least 6 months were included; however, studies in which intervention was given for supplementary and therapeutic purposes were excluded. Recommendations are made for input to the Lives Saved Tool (LiST) model by following standardized guidelines developed by Child Health Epidemiology Reference Group (CHERG).

**Results:**

We included 16 studies in this review. Amongst these, 9 studies provided education on complementary feeding, 6 provided complementary feeding (with our without education) and 1 provided both as separate arms. Overall, education on CF alone significantly improved HAZ (SMD: 0.23; 95% CI: 0.09, 0.36), WAZ (SMD 0.16, 95% CI: 0.05, 0.27), and significantly reduced the rates of stunting (RR 0.71; 95% CI: 0.56, 0.91). While no significant impact were observed for height and weight gain. Based on the subgroup analysis; ten studies from food secure populations indicated education on CF had a significant impact on height gain, HAZ scores, and weight gain, however, stunting reduced non-significantly. In food insecure population, CF education alone significantly improved HAZ scores, WAZ scores and significantly reduced the rates of stunting, while CF provision with or without education improved HAZ and WAZ scores significantly.

**Conclusion:**

Complementary feeding interventions have a potential to improve the nutritional status of children in developing countries. However, large scale high quality randomized controlled trials are required to assess the actual impact of this intervention on growth and morbidity in children 6-24 months of age. Education should be combined with provision of complementary foods that are affordable, particularly for children in food insecure countries.

## Background

About one third of deaths in children under 5 years of age are due to underlying undernutrition, which includes stunting, severe wasting, deficiencies of vitamin A and zinc, and suboptimum breastfeeding [1]. Childhood malnutrition is prevalent in low and middle income countries (LMICs), and according to an estimate, 19.4% of children <5 years of age in these countries were underweight (weight-for-age Z score <-2) and about 29.9% were stunted (height-for-age Z score <-2) in the year 2011 [2]. The prevalence of both underweight and stunting is highest in Africa and South-Central Asia and stunting and wasting along with intrauterine growth restriction are responsible for about 2.1 million deaths worldwide in children <5 years of age [3].

Infants from 6 to 18 months are especially vulnerable to developing malnutrition. In order to sustain the gains made by promoting exclusive breastfeeding for the first six months of life, interventions need to extend into the second half of infancy and beyond. This could be ensured by enabling caregivers to appropriately feed their children with safe and adequate complementary foods while maintaining frequent breastfeeding [4]. It is well recognized that the period of 6-24 months of age is one of the most critical time in the growth of an infant [5]. The incidence of stunting is the highest in this period as children have high demand for nutrients and there are limitations in the quality and quantity of available foods, especially after exclusive breastfeeding [1,6]. Complementary feeding (CF) for infants refers to the timely introduction of safe and nutritional foods in addition to breast-feeding i.e. clean and nutritionally rich additional foods introduced at about six months of infant age [5]. According to the World Health Organization, CF should be timely, adequate, appropriate, and given in sufficient quantity [7].

Several strategies have been employed to improve CF practices [1]. These include nutritional counseling to mothers designed to promote healthy feeding practices; provision of CF offering extra energy (with or without micronutrient fortification); and increasing energy density of complementary foods through simple technology [1,8].

Over the last five years, several reviews have been published on the impact of various CF interventions [1,5,9]. Dewey and Adu-Afarwah 2008 [1] included seven efficacy trials which indicated that provision of CF can have a significant impact on growth under well-controlled situations. Imdad et al. [5] demonstrated that both provision of appropriate CF (with or without nutrition counseling) and nutrition counseling alone have a significant impact on improving weight and linear growth.

In this review, we have assessed the impact of education on CF and provision of CF with or without education on growth and morbidity among children under 2 years of age in LMICs. We reviewed the available literature and evaluated the quality of included studies according to the Child Health Epidemiology Reference Group (CHERG) adaptation of Grading of Recommendations, Assessments, Development and Education (GRADE) criteria [10].

## Methods

To evaluate the impact of CF interventions on child growth, a search, following CHERG Systematic Review Guidelines, was conducted on PubMed, Cochrane Library, Google Scholar and WHO global database. We also reviewed the reference lists of identified articles, existing reviews and meta-analyses, and looked for studies that were not identified in the initial search. We included randomized (individual or cluster) and non-randomized controlled trials and programs. The last date of search was October 01, 2012 and we did not apply any language restriction.

### Inclusion/exclusion criteria

Studies on education on CF and provision of CF (fortified or unfortified, but not micronutrients alone) with or without education were included if those provided interventions for at least 6 months and were conducted in LMICs on children less than 2 years of age. Studies in which interventions were given for supplementary and therapeutic purposes were excluded. We also excluded studies in which fortified complementary foods were compared with non-fortified complementary foods.

The developing countries were defined according to World Bank classification [11]. Studies were classified into one of two groups according to the food security status of the study populations. Studies in populations with an average per capita income under USD 1.25 were classified as “food insecure” while studies in populations with a higher income were classified as “food secure”. The primary outcomes were height gain (cm), weight gain (kg), the absolute gain in height-for-age Z score (HAZ) and weight-for-age Z score (WAZ), stunting (<-2 HAZ) and underweight (<-2 WAZ). We also studied outcomes for morbidity including diarrhea, respiratory infections and fever.

### Data abstraction

For the studies that met the final inclusion criteria, data was abstracted describing study identifiers and context, study design and limitations, intervention specifics and outcome effects into a standardized abstraction form as detailed in the CHERG Systematic Review Guidelines [10]. Each study was assessed and graded according to the CHERG adaptation of the GRADE technique. Randomized trials received an initial score of “high”. One- to two-point grade increases were allotted to studies with statistically significant strong levels of association (>80% reduction).

### Quantitative data synthesis

We conducted meta-analyses for individual studies and pooled statistics was reported as the relative risk (RR) for categorical data and standard mean difference (SMD) for continuous data between the experimental and control groups with 95% confidence intervals (CIs). HAZ scores were further converted into stunting considering the standard deviation of 1.4 for stunting in the population [9]. All analyses were conducted using the software Review Manager 5.1. Heterogeneity was quantified by Chi^2^ and I^2^, which can be interpreted as the percentage of the total variation between studies that is attributable to heterogeneity rather than to chance, a low p-value (less than 0.1) or a large chi-squared statistic relative to its degree of freedom and I^2^ values greater than 50% were taken as substantial and high heterogeneity. A subgroup analysis was also performed based on food security of the populations.

We summarized the evidence by outcome, including qualitative assessments of study quality and quantitative measures, according to the standard guidelines. A grade of “high”, “moderate”, “low” and “very low” was used for grading the overall evidence indicating the strength of an effect on specific health outcome according to the CHERG Rules for Evidence Review[10]. The study characteristics for the impact of education on complementary feeding are shown in additional file 1. The study characteristics for studies that investigated the impact of the provision of complementary feeding with or without education are presented in additional file 2.

## Results

**Figure 1:**
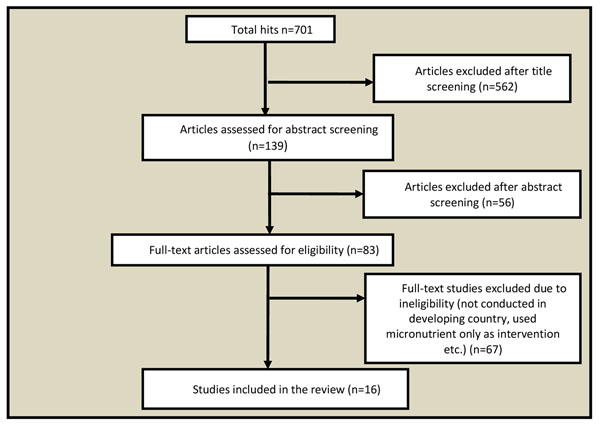
Search flow diagram

We found 701 titles on the initial search, of which 16 met the inclusion criteria and were finally included (figure 1). Amongst these, ten studies provided education on CF [12-21] alone whereas, seven [17,22-27] provided CF with or without education. Bhandari et al [17] was a three arm study in which one arm received education on CF and the second arm received CF, while the third arm was a control group with no intervention. Six of the included studies [13-15,19-21] were from food insecure population, while ten were from food secure.

### Overall impact

#### Education on CF

**Figure 2:**
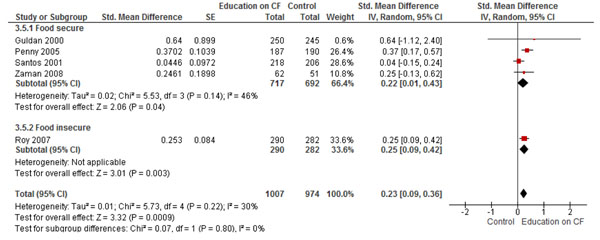
Forest Plot for the effect of education on complementary feeding and HAZ scores: Food secure vs. insecure population

**Figure 3:**
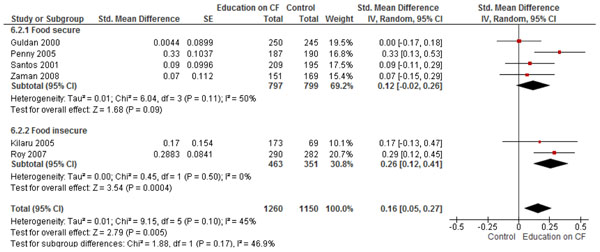
Forest Plot for the effect of education on complementary feeding and WAZ scores: Food secure vs. food insecure population

**Figure 4:**
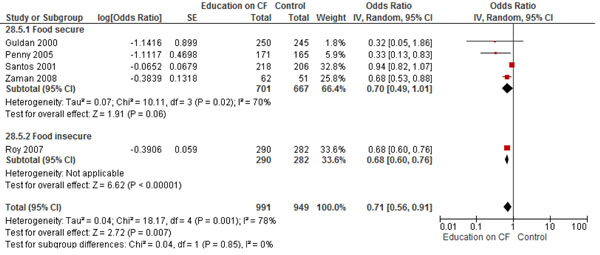
Forest Plot for the effect of education on complementary feeding and stunting: Food secure vs. insecure population

The combined pooled analysis of studies from both food secure and insecure populations, found that education on CF alone significantly improved HAZ (SMD: 0.23; 95% CI: 0.09, 0.36, 5 studies) (Figure 2), WAZ (SMD 0.16, 95% CI: 0.05, 0.27, 6 studies) (Figure 3), and significantly reduced the rates of stunting (RR 0.71; 95% CI: 0.60, 0.76, 5 studies) (Figure 4). While no significant impact were observed for height gain (SMD 0.23; 95% CI: -0.00, 0.45, 6 studies) and weight gain (SMD 0.26; 95% CI: -0.00, 0.52, 7 studies). Education on CF alone was also assessed to evaluate its effect on improving the actual feeding practices or compliance with the recommendations. We evaluated compliance by pooling the average uptake of various recommended foods and found that education on CF significantly improved the uptake of these recommended foods by 62% (RR: 1.62, 95% CI: 1.17-2.26).

#### Provision of CF with or without education

**Figure 5:**
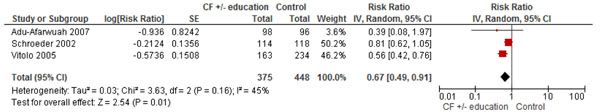
Forest Plot for the effect of complementary feeding with or without education: Acute respiratory infections

The combined pooled analysis indicated that provision of CF with or without education reduced the incidence of respiratory infections by 33% (95% CI: 0.49, 0.91, 3 studies) (Figure 5). However, no significant impacts were observed on diarrhea (RR 0.75; 95% CI: 0.42, 1.35, 3 studies) and fever (RR 0.89; 95% CI: 0.41, 1.90, 2 studies).

### Food secure population

#### Education on CF

We found six studies that evaluated education on CF alone [13-15,19-21]. The pooled analysis found significant impact of education on CF on height gain (SMD 0.35; 95% CI: 0.08, 0.62, 4 studies), HAZ scores (SMD 0.22; 95% CI: 0.01, 0.43, 4 studies) (Figure 2) and weight gain (SMD 0.40; 95% CI: 0.02, 0.78, 4 studies), while no significant impact was observed for WAZ scores (SMD 0.12; 95% CI: -0.02, 0.26, 4 studies) (Figure 3) and stunting (RR 0.70; 95% CI: 0.49 1.01, 4 studies) (Figure 4).

### Food insecure population

#### Education on CF

We found four studies from food insecure population that provided education on complementary feeding alone [12,16-18]. The pooled analysis found a significant impact on HAZ (SMD 0.25; 95% CI: 0.09, 0.42, 1 study) (Figure 2), WAZ scores (SMD 0.26; 95% CI: 0.12, 0.41, 2 studies) and significantly reduced the rates of stunting (RR 0.68; 95% CI: 0.60, 0.76, 1 study) (Figure 3 and 4). While there was no significant impact observed on height gain (SMD 0.00; 95% CI: -0.15, 0.16, 2 studies), weight gain (SMD 0.06; 95% CI: -0.13, 0.25, 3 studies), and underweight (RR 1.03; 95% CI: 0.90, 1.18, 1 study).

#### Provision of CF with or without education

**Figure 6:**
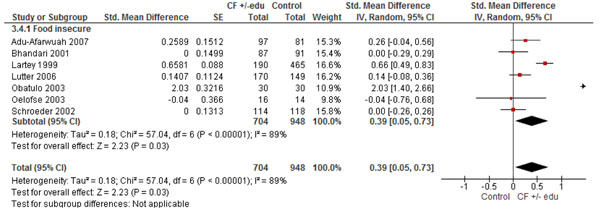
Forest Plot for the effect of complementary feeding with or without education on HAZ scores: Food insecure population

**Figure 7:**
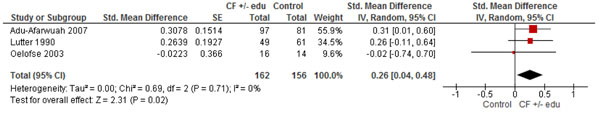
Forest Plot for the effect of complementary food with or without education: WAZ scores: Food insecure population

We identified 7 studies [17,22-27] that provided CF with or without education in food insecure population. The pooled analyses found a significant impact on HAZ (SMD 0.39; 95% CI: 0.05, 0.73, 7 studies) (Figure 6), WAZ scores (SMD 0.26; 95% CI: 0.04, 0.48, 3 studies) (figure 7), and significantly reduced underweight (RR 0.35; 95% CI: 0.16, 0.77, 1 study). However, non-significant impacts were observed for height gain (SMD 0.34; 95% CI: -0.09, 0.78, 4 studies), weight gain (SMD 0.43, 95% CI: -0.42, 1.27, 4 studies) and stunting (RR 0.33; 95% CI: 0.11, 1.00, 7 studies).

### Recommendation for the LiST model

Based on the volume and consistency of the evidence, the quality of available evidence was assessed to be that of ‘moderate’ level (Table 1, 2, and 3). For input to Lives Saved Tool (LiST), we applied the CHERG rules for evidence review to the outcomes assessed for the impact of CF and education on complementary feeding on growth of children less than 2 years of age. As there was no data on mortality; either all-cause or cause specific, we used the rates of stunting to estimate that provision of CF with or without education will result in a 67% reduction in stunting in food insecure populations while education on complementary feeding alone will result in a 30% reduction in stunting in food secure population. These recommendations are for children aged 6-24 months.

**Table 1:** Quality assessment of trials on nutrition education

	Quality Assessment					Summary of Findings		
				**Directness**		**No of events**		

**No of studies**	**Design**	**Limitations**	**Consistency**	**Generalizability to population of interest**	**Generalizability to intervention of interest**	**Intervention**	**Control**	**RR or SMD (95% CI)**

***Height gain: Moderate outcome-specific quality***								

6 studies **Food secure**[13,15,19,21] **Food insecure**[16,17]	RCT + non RCT	Random effect model was used because of heterogeneity	3 studies suggest benefit			1410	1327	SMD 0.35 (0.08, 0.62) **Food secure population** SMD 0.00 (-0.15, 0.16) ***Food insecure population***

***Height for age: Moderate outcome-specific quality***								

5 studies **Food secure**[14,15,19,21] **Food insecure**[18]	RCT + non RCT	Random effect model was used because of heterogeneity	2 studies suggest benefit			1007	974	SMD 0.22 (0.01, 0.43) ***Food secure population*** SMD 0.25 (0.09, 0.42) ***Food insecure population***

***Stunting: Moderate outcome-specific quality***								

**Food secure**[14,15,19,21] **Food insecure**[18]	RCT + non RCT	Random effect model was used because of heterogeneity	1 study suggest benefit			991	949	RR 0.70 (0.49,1.01) ***Food secure population*** RR 0.68 (0.60,0.76) ***Food insecure population***

***Weight gain: Moderate outcome-specific quality***								

7 studies **Food secure**[13,15,19,21] **Food insecure**[12,16,17]	RCT + non RCT	Random effect model was used because of heterogeneity	4 studies suggest benefit			1583	397	SMD 0.40 (0.02, 0.78) ***Food secure population*** SMD 0.06 (-0.13, 0.25) ***Food insecure population***

***Weight-for-age: Moderate outcome-specific quality***								

6 studies **Food secure**[14,15,19,21] **Food insecure**[12,18]	RCT + non RCT	Random effect model was used because of heterogeneity	2 studies suggest benefit			1260	1150	SMD 0.12 (-0.02, 0.26) ***Food secure population*** SMD 0.26 (0.12, 0.41) ***Food insecure population***

***Underweight: Moderate outcome-specific quality***								

1 study[16]	RCT	Fixed effects		Only one study and to food secure population		435	394	RR 1.03 (0.90, 1.18) **Food secure population**

**Table 2:** Quality assessment of trials on complementary food with or without education

	Quality Assessment					Summary of Findings		
				**Directness**		**No of events**		

**No of studies**	**Design**	**Limitations**	**Consistency**	**Generalizability to population of interest**	**Generalizability to intervention of interest**	**Intervention**	**Control**	**RR or SMD (95% CI)**

***Height gain: Moderate outcome-specific quality***								

4 studies[17,24-26]	RCT	Random effect model was used because of heterogeneity	2 studies suggest benefit	To food insecure population		257	255	SMD 0.34(-0.09, 0.78) ***Food insecure population***

***Height for age: Moderate outcome-specific quality***								

7 studies[17,22,24-26,29,30]	RCT +non RCT	Random effect model was used because of heterogeneity	2 studies suggest benefit	To food insecure population		704	948	SMD 0.39 (0.05, 0.73) ***Food insecure population***

***Stunting: Moderate outcome-specific quality***								

7 studies[17,22,24-26,29,30]	RCT +non RCT	Random effect model was used because of heterogeneity		To food insecure population		704	948	RR 0.33 (0.11, 1.00) ***Food insecure population***

***Weight gain: Moderate outcome-specific quality***								

4 studies[17,24-26]	RCT	Random effect model was used because of heterogeneity	1 study suggest benefit	To food insecure population		247	255	SMD 0.43 (-0.42, 1.27) **Food insecure population**

***Weight-for-age: Moderate outcome-specific quality***								

3 studies[22,25,30]	RCT+ non RCTs	Random effect model was used because of heterogeneity	1 study suggest benefit	To food insecure population		162	156	SMD 0.26 (0.04, 0.48) **Food insecure population**

***Underweight: Moderate outcome-specific quality***								

1 study[30]	Non RCT			Only one study and to food insecure population		170	149	RR 0.35 (0.16, 0.77) **Food insecure population**

**Table 3:** Quality assessment of trials on complementary food with or without education

	Quality Assessment					Summary of Findings		
				**Directness**		**No of events**		

**No of studies**	**Design**	**Limitations**	**Consistency**	**Generalizability to population of interest**	**Generalizability to intervention of interest**	**Intervention**	**Control**	**RR or SMD (95% CI)**

***Respiratory infections: Moderate outcome-specific quality***								

3 studies [20,22,26]	RCT	Random effect model was used because of heterogeneity	One study suggest benefit			375	448	RR 0.67 (0.49, 0.91)

***Diarrhea/vomiting: Moderate outcome-specific quality***								

3 studies [20,22,26]	RCT	Random effect model was used because of heterogeneity	None of the study suggest benefit			424	488	RR 0.75 (0.42, 1.35)

***Fever: low outcome-specific quality***								

2 studies [17,22]	RCT	Random effect model was used because of heterogeneity	None of the study suggest benefit			195	187	RR 0.89 ( 0.41, 1.90)

## Discussion

Despite clear evidence of the disastrous consequences of childhood nutritional deprivation in the short and long terms, nutritional health remains a low priority. Therefore, enhanced and rigorous actions are needed to deliver and scale up education and provision of complementary feeding interventions. In this review we have included trials that evaluated the disaggregated evidence of the impact of education on CF alone, and provision of CF with or without education (excluding those on food fortification and supplementary feeding) on growth and morbidity in children less than 2 years of age in LMICs.

Our review indicates that in food secure population, education on CF had a significant impact on linear growth as evident by significant increase in height gain and HAZ scores, and also significantly improved weight gain, however rates of stunting reduced non-significantly. Education in food insecure population also improved linear growth and weight gain as evident by significant increase in HAZ and WAZ scores and significant decrease in rates of stunting. We did not find any study on provision of complementary feeding (with or without education) from food secure population, however, from food insecure population the intervention improved HAZ and WAZ scores. Our review indicated that CF provision had no significant impacts on height or weight gain, while previous reviews [5,9] suggested otherwise, an explanation for this could be that these reviews also included studies that provided the intervention for less than 6 months, Imdad et al. [5], on the other hand, also included studies on children with moderate malnutrition.

As part of causal chain, it is well recognized that educational interventions improve feeding practices which then lead to improved growth outcomes. The educational messages should lay emphasis on the importance of appropriate home prepared foods, hygiene and high energy foods and it is important to assess the recall of the messages by mothers once the messages are delivered and our review suggest a significant 62% increase in compliance with the imparted messages, reinforcing the importance of such intervention. Considerable variations were observed in the types of educational messages delivered and an attempt to assess the quality of educational messages and delivery strategies was difficult, but in general most of the studies delivered educational interventions of reasonably good quality with the appropriate use of charts, posters and booklets. The two studies that had the most impact on linear growth [15,21], also provided clear messages regarding the use of affordable home-prepared animal source products which indicates that giving messages specifically promoting the use of nutrient-rich animal products may have an impact on growth. However, financial constraints limit the possibility of including adequate amount of animal products in the child's diet, particularly among food insecure populations. Thus, in food insecure populations these nutritional messages need to be combined with provision of adequate amounts of animal products. One option can be the use of protein-rich plant foods, however, most plant foods, especially staples, legumes, lentils and vegetables contain anti-nutrients which can reduce the bioavailability of micronutrients and interfere with digestion. These include phytate and alpha amylase. Processing is required in order to reduce the content of anti-nutrients such as phytate or addition of alpha amylase in order to increase the impact of plant foods. This is in turn associated with additional cost and required expertise.

Nutritional status has a strong and consistent relation to death from respiratory infections. Nutrition education and complementary feeding with or without education had a positive impact on reducing respiratory infections. A review by Rice et al. [28] reported that the risk of mortality from respiratory infections is increased by two folds to three folds if associated with anthropometric status. Respiratory infections are one of the leading killers of children in developing countries. Prevention of undernutrition can potentially have an indirect impact on reducing childhood mortality through respiratory infections.

There were a variety of complementary food(s) used as intervention in the included studies. Amongst these foods were maize, fortified fat based spread, food prepared from locally available raw ingredients, cereal and porridge. The scarcity of available studies and their heterogeneity as well as the variety in complementary feeding interventions makes it difficult to conclude one particular type of complementary feeding intervention as the most effective; moreover, the variation in the reported outcomes amongst studies makes it difficult to compare them.

In future, further trials are needed particularly from food insecure population in which interventions are consistent and standardized in terms of study design and quality, complementary food chosen, duration of intervention and should report consistent outcomes for growth and morbidity. However, the available evidence is sufficient to recommend that in food insecure populations, education should be accompanied with provision of affordable yet effective complementary food. Accelerated and concerted actions are required to deliver and scale up nutrition education and CF provision interventions that are cost-effective, feasible and effective in improving the nutritional status of children.

## Conclusion

CF interventions have a high potential to improve the nutritional status of children in developing countries. Nutrition education interventions should be combined with provision of CF that are affordable, particularly in food insecure countries.

## List of abbreviations used

Child Health Epidemiology Reference Group (CHERG); confidence interval (CI); Grading of Recommendations, Assessments, Development and Education (GRADE); Height-for-age Z score (HAZ); Low and middle income countries (LMICs); Randomized Controlled Trials (RCTs); Relative risk (RR); Standard deviation (SD); Standard mean difference (SMD); Weighted mean differences (WMD); Weight-for-age Z score (WAZ).

## Competing interests

We do not have any financial or non-financial competing interests for this review.

## Authors' contributions

Dr. ZAB was responsible for designing and co-ordinating the review. ZSL, GZ and JKD were responsible for: data collection, screening the search results, screening retrieved papers against inclusion criteria, appraising quality of papers, abstracting data from papers, entering data into RevMan, analysis and interpretation of data. ZSL, GZ, JKD and ZAB wrote the paper. ZAB and ZSL critically reviewed and modified the manuscript.

## Supplementary Material

Additional File 1 Characteristics of studies on impact of education on complementary feeding. Click here for file

Additional File 2 Characteristics of studies on impact of provision of complementary feeding with or without education. Click here for file

## Declaration

The publication costs for this supplement were funded by a grant from the Bill & Melinda Gates Foundation to the US Fund for UNICEF (grant 43386 to "Promote evidence-based decision making in designing maternal, neonatal, and child health interventions in low- and middle-income countries”). The Supplement Editor is the principal investigator and lead in the development of the Lives Saved Tool (LiST), supported by grant 43386. He declares that he has no competing interests.

This article has been published as part of *BMC Public Health* Volume 13 Supplement 3, 2013: The Lives Saved Tool in 2013: new capabilities and applications. The full contents of the supplement are available online at http://www.biomedcentral.com/bmcpublichealth/supplements/13/S3.

## References

[B1] Dewey KG Adu-Afarwuah S Systematic review of the efficacy and effectiveness of complementary feeding interventions in developing countries Maternal Child Nutrition 2008 4 Suppl 1 24 85 18289157 10.1111/j.1740-8709.2007.00124.x PMC6860813

[B2] Stevens GA Finucane MM Paciorek CJ Flaxman SR White RA Donner AJ Ezzati M Trends in mild, moderate, and severe stunting and underweight, and progress towards MDG 1 in 141 developing countries: a systematic analysis of population representative data Lancet 2012 380 824 834 10.1016/S0140-6736(12)60647-3 22770478 PMC3443900

[B3] Black RE Allen LH Bhutta ZA Caulfield LE de Onis M Ezzati M Mathers C Rivera J Maternal and child undernutrition: global and regional exposures and health consequences Lancet 2008 371 9608 243 260 10.1016/S0140-6736(07)61690-0 18207566

[B4] WHO Implementing the global strategy for infant and young child feeding 2003 Geneva: World Health Organization Available at http://whqlibdoc.who.int/publications/2003/924159120X.pdf

[B5] Imdad A Yakoob MY Bhutta ZA Impact of maternal education about complementary feeding and provision of complementary foods on child growth in developing countries BMC Public Health 2011 11 Suppl 3 S25 10.1186/1471-2458-11-S3-S25 21501443 PMC3231899

[B6] Shrimpton R Victora CG de Onis M Lima RC Blössner M Clugston G Worldwide timing of growth faltering: implications for nutritional interventions Pediatrics 2001 107 5 E75 10.1542/peds.107.5.e75 11331725

[B7] WHO Report of Informal Meeting to Review and Develop Indicators for Complementary Feeding http://whqlibdoc.who.int/hq/2002/a91059.pdf Food and Nutrition Program Regional Office for the Americas 2002 Washington, D.C: World Health Organization

[B8] Caulfield LE Huffman SL Piwoz EG Interventions to improve intake of complementary foods by infants 6 to 12 months of age in developing countries: impact on growth and on the prevalence of malnutrition and potential contribution to child survival Food Nutr Bull 1999 20 183 200

[B9] Bhutta ZA Ahmed T Black RE Cousens S Dewey K Giugliani E Haider BA Kirkwood B Morris SS Sachdev HPS Shekar M Maternal and Child Undernutrition Study Group What works? Interventions for maternal and child undernutrition and survival Lancet 2008 371 9610 417 440 10.1016/S0140-6736(07)61693-6 18206226

[B10] Walker N Fischer-Walker C Bryce J Bahl R Cousens S Standards for CHERG reviews of intervention effects on child survival International journal of epidemiology 2010 39 suppl 1 i21 i31 20348122 10.1093/ije/dyq036 PMC2845875

[B11] 2012 list of developing countries 2011 World Bank Available at http://web.worldbank.org/, accessed on June 21, 2012

[B12] Kilaru A Griffiths PL Ganapathy S Shanti G Community-based nutrition education for improving infant growth in rural Karnataka Indian Pediatr 2005 42 5 425 15923688

[B13] Shi L Zhang J Wang Y Caulfield LE Guyer B Effectiveness of an educational intervention on complementary feeding practices and growth in rural China: a cluster randomised controlled trial Public Health Nutr 2009 13 04 556 565 19706219 10.1017/S1368980009991364

[B14] Zaman S Ashraf RN Martines J Training in complementary feeding counselling of healthcare workers and its influence on maternal behaviours and child growth: a cluster-randomized controlled trial in Lahore, Pakistan J Health Popul Nutr 2008 26 2 210 18686554 PMC2740673

[B15] Penny ME Creed-Kanashiro HM Robert RC Narro MR Caulfield LE Black RE Effectiveness of an educational intervention delivered through the health services to improve nutrition in young children: a cluster-randomised controlled trial Lancet 2005 365 9474 1863 1872 10.1016/S0140-6736(05)66426-4 15924983

[B16] Bhandari N Mazumder S Bahl R Martines J Black RE Bhan MK An educational intervention to promote appropriate complementary feeding practices and physical growth in infants and young children in rural Haryana, India J Nutr 2004 134 9 2342 2348 15333726 10.1093/jn/134.9.2342

[B17] Bhandari N Bahl R Nayyar B Khokhar P Rohde JE Bhan MK Food supplementation with encouragement to feed it to infants from 4 to 12 months of age has a small impact on weight gain J Nutr 2001 131 7 1946 1951 11435512 10.1093/jn/131.7.1946

[B18] Roy SK Jolly SP Shafique S Fuchs GJ Mahmud Z Chakraborty B Roy S Prevention of malnutrition among young children in rural Bangladesh by a food-health-care educational intervention: a randomized, controlled trial Food Nutr Bull 2007 28 4 375 383 18274163 10.1177/156482650702800401

[B19] Santos I Victora CG Martines J Goncalves H Gigante DP Valle NJ Pelto G Nutrition counseling increases weight gain among Brazilian children J Nutr 2001 131 11 2866 2873 11694610 10.1093/jn/131.11.2866

[B20] Vitolo MR Bortolini GA Feldens CA de Lourdes M Drachler Impactos da implementacao dos dez passos da alimentacao saudavel para criancas: ensaio de campo randomizado: Impacts of the 10 Steps to Healthy Feeding in Infants: a randomized field trial Cad Saude Publica 2005 21 5 1448 1457 10.1590/S0102-311X2005000500018 16158151

[B21] Guldan GS Fan HC Ma X Ni ZZ Xiang X Tang MZ Culturally appropriate nutrition education improves infant feeding and growth in rural Sichuan, China J Nutr 2000 130 5 1204 1211 10801920 10.1093/jn/130.5.1204

[B22] Adu-Afarwuah S Lartey A Brown KH Zlotkin S Briend A Dewey KG Randomized comparison of 3 types of micronutrient supplements for home fortification of complementary foods in Ghana: effects on growth and motor development Am J Clin Nutr 2007 86 2 412 420 17684213 10.1093/ajcn/86.2.412

[B23] Lutter CK Rodraguez A Fuenmayor G Avila L Sempertegui F Escobar J Growth and micronutrient status in children receiving a fortified complementary food J Nutr 2008 138 2 379 388 18203907 10.1093/jn/138.2.379

[B24] Obatolu VA Growth pattern of infants fed with a mixture of extruded malted maize and cowpea Nutrition 2003 19 2 174 178 10.1016/S0899-9007(02)01102-4 12591556

[B25] Oelofse A Van Raaij JMA Benade AJS Dhansay MA Tolboom JJM Hautvast J The effect of a micronutrient-fortified complementary food on micronutrient status, growth and development of 6-to 12-month-old disadvantaged urban South African infants Int J Food Sci Nutr 2003 54 5 399 407 10.1080/0963748031000092161 12907410

[B26] Schroeder DG Pachón H Dearden KA Kwon CB Ha TT Lang TT Marsh DR An integrated child nutrition intervention improved growth of younger, more malnourished children in northern Viet Nam Food Nutr Bull 2002 23 4 Suppl 53 61 12503232

[B27] Lartey A Manu A Brown KH Peerson JM Dewey KG A randomized, community-based trial of the effects of improved, centrally processed complementary foods on growth and micronutrient status of Ghanaian infants from 6 to 12 mo of age Am J Clin Nutr 1999 70 3 391 404 10479202 10.1093/ajcn/70.3.391

[B28] Rice AL Sacco L Hyder A Black RE Malnutrition as an underlying cause of childhood deaths associated with infectious diseases in developing countries Bull World Health Organ 2000 78 10 1207 1221 11100616 PMC2560622

[B29] Lartey A Manu A Brown KH Peerson JM Dewey KG A randomized, community-based trial of the effects of improved, centrally processed complementary foods on growth and micronutrient status of Ghanaian infants from 6 to 12 mo of age Am J Clin Nutr 1999 70 3 391 404 10479202 10.1093/ajcn/70.3.391

[B30] Lutter CK Rodraguez A Fuenmayor G Avila L Sempertegui F Escobar J Growth and micronutrient status in children receiving a fortified complementary food J Nutr 2008 138 2 379 388 18203907 10.1093/jn/138.2.379

