# Supplementary material for: Impact of education and provision of complementary feeding on growth and morbidity in children less than 2 years of age in developing countries: a systematic review
Source: BMC Public Health. 2013 Sep 17;13(Suppl 3):S13. doi: 10.1186/1471-2458-13-S3-S13 (PMC3847349; doi:10.1186/1471-2458-13-S3-S13)
Supplement: Additional File 1 — Characteristics of studies on impact of education on complementary feeding. [file 1471-2458-13-S3-S13-S1.docx]

| **Additional file 1: chacteristics of included studies – nutritional education only** | | | | | | | | | | | | | | |  |  |
| --- | --- | --- | --- | --- | --- | --- | --- | --- | --- | --- | --- | --- | --- | --- | --- | --- |
| **Author** | **Country** | **Region** | **Type of study** | **Age group/ sample size** | **Intervention- what was the educational message** | **Control** | **Who delivered** | **Where was the education given** | **Duration of intervention** | **Baseline demographics** | **Baseline nutritional status** | **Seasonal variation** | **Duration of follow up** | **Results** |  |  |
| Shi 2009^18^ | China | Rural | cRCT  Effectiveness  Food secure | 2-4 mo/  I: 294  C: 305 | Educational messages and enhanced home-prepared recipes were disseminated to caregivers through group trainings and home visits.  Messages: (i) group training sessions on food selection, preparation and hygiene, childhood nutrition and growth, and responsive feeding style; demonstrated of preparing enhanced weaning food recipes which were  formulated using locally available, affordable, acceptable and nutrient-dense foods such as egg, tomato, beans, meat, chicken and liver; (iii) booklets which contained  infant feeding guidance and methods of preparing the  recommended recipes; and (iv) home visits every three  months to identify possible feeding problems and provide  Individual counselling. | received a standard package of child  health care from the township hospitals which included  breast-feeding counselling, but did not contain other than  standard counselling on complementary feeding. | Health-care providers | Health facility & home visits | 12 months | Infants in the intervention group did not differ significantly from controls However, more mothers at intervention sites than controls engaged in agriculture work (57.1% vs. 49.8%) and more fathers at intervention sites than controls were migrant labourers who worked temporarily in cities (67.3% v. 55.7%). | At baseline,  there were no significant differences in the mean weights  and lengths between the intervention and control groups  (Weight: 6?51 v. 6?66 kg, P50?09; length: 60?75 v.  61?10 cm, P50?17). | - | 8-10 mo | Adjusted difference: weight (kg) gain Mean (95% CI) 0.22 (0.003, 0.45)  Length gain (cm) 0.66 (0.03, 1.29) |  |  |
| **Author** | **Country** | **Region** | **Type of study** | **Age group** | **Intervention- what was the educational message** | **Control** | **Who delivered** | **Where was the education given** | **Duration of intervention** | **Baseline demographics** | **Baseline nutritional status** | **Seasonal variation** | **Duration of follow up** | **Results** |  |  |
| Zaman 2008^20^ | Pakistan | Urban | RCT  Efficacy  Food secure | 6-24 mo/  I: 151  C: 169 | Educational: training health workers in nutrition counseling using The Integrated Management of Childhood Illness IMCI Module-Counsel the mother. | No intervention | IMCI module module—‘Counsel the Mother was used. A local adaptation of Pakistan’s IMCI ‘feeding counselling card’ was developed in the local language. | Community centre | No precise information given. Lady health visitors were trained to deliver education to mothers when they visited health centers. These children were followed up till 180 days | The socioeconomic and demographic characteristics  of the two groups were similar | - | - | 180 days | Intervention grp: WAZ 12+ mths: -0.35 +-0.947  HAZ: -0.35+- 0.947    Control: WAZ  0.814+- 1.02  HAZ -0.814+- 1.02 | |  |
| Penny 2005^21^ | Peru | Peri-urban | cRCT  Effectiveness  Food secure | Newborn/  I: 187  C: 190 | Health staff received education in counseling and anthropometry; high-performing facilities were accredited. 3 key messages:  a. Use thick purees instead of soups and at each meal give puree first b. Add a special food to your baby’s serving (e.g. chicken liver, egg or fish) c. Teach your child to eat with love, patience and good humour | No education | Health facility staff | Health facility | Nutrition eduction was given to care givers duing health facility visit and these children were followed till 18 months | Baseline characteristics in terms of SES were similar between the two groups; except that the intervention group has slightly better maternal education and hygiene score. | There were only slightly differences in birth weight between the intervention and control groups at baseline (mean 3.41 vs. 3.35 kg) and no differences in length (cm). | - | 18 mo | Intervention grp:  WAZ: -0.33+-0.90  HAZ: -0.81+-0.80    Control:  WAZ: -0.62+-0.83  HAZ  : -1.19+-0.83 | |  |
| **Author** | **Country** | **Region** | **Type of study** | **Age group** | **Intervention- what was the educational message** | **Control** | **Who delivered** | **Where was the education given** | **Duration of intervention** | **Baseline demographics** | **Baseline nutritional status** | **Seasonal variation** | **Duration of follow up** | **Results** | |  |
| Vitolo 2005^22^ | Brazil | Unclear | cRCT  Efficacy  Food secure | 0-12 mo  N=397 | Mothers received educational guidelines for infant and child feeding from birth to 1 year post-partum through home visits. Based on ‘Ten Steps to Healthy Feeding’:  a. Feed only breastmilk for up to 6 months  b. Gradually introduce other foods after 6 months while maintaining breastfeeding  c. Give CF 3¥ per day after 6 months  d. Ensure that no schedules impair the offering of CF  e. Offer ‘thick’ foods using spoons  f. Offer child different foods during the day  g. Stimulate daily consumption of fruits/vegetables  h. Avoid sugar and other junk foods  i. Pay attention to hygiene and proper handling of food  j. Stimulate sick/convalescent to eat | No intervention | Community-based  nutrition educators | Homes | 10 home visits,  performed in the first 10 days after  parturition and then monthly to 6 month  at 8, 10 and 12 months. |  |  |  | 12 months | Intervention grp: % Anemia: 66.2    Control: % Anemia: 61.8 | |  |
| **Author** | **Country** | **Region** | **Type of study** | **Age group** | **Intervention- what was the educational message** | **Control** | **Who delivered** | **Where was the education given** | **Duration of intervention** | **Baseline demographics** | **Baseline nutritional status** | **Seasonal variation** | **Duration of follow up** | **Results** | |  |
| Bhandari 2004^24^ | India | Rural | cRCT  Efficacy  Food secure | Newborns (10 d or younger)/  I: 552  C: 473 | Mothers received education on food preparation, food diversity and use of amylase rich flour.  • Health and nutrition workers in Intervention communities were trained (for 3 d) in age appropriate complementary feeding (immediate before after birth, exclusive before 4-6 mo, initiate complementary feeding 4-6 mo, education on types of food to feed, frequency of feeding, amount, child encouragement, hand washing, feeding during illness)  • Health and nutrition workers in Control Communities did not receive any specific training or information | No intervention | health/nutrition workers | Health facility | 12 months | The baseline characteristics of the children enrolled in the cohort in the intervention and control communities were similar,  except for the proportion of mothers working outside home, which was higher in the intervention communities | Both groups had similar birth weights at baseline. | - | 18 months | Intervention group: weight gain (kg):  1.16+-0.65  length gain (cm): 6.01+-2.01  % underweight: 54.2  % stunted: 50.1    Control: weight gain (kg): 1.15+-0.67  Length gain (cm): 5.91+-1.83  %  underweight: 52.9  % stunted: 51.2 | |  |
| **Author** | **Country** | **Region** | **Type of study** | **Age group** | **Intervention- what was the educational message** | **Control** | **Who delivered** | **Where was the education given** | **Duration of intervention** | **Baseline demographics** | **Baseline nutritional status** | **Seasonal variation** | **Duration of follow up** | **Results** | | |
| Santos 2001^26^ | Brazil | Urban | cRCT  Efficacy  Food secure | < 18 mo/  I: 209  C: 195 | Health-care providers were trained to deliver educational messages on food preparation and infant feeding to mothers. Key messages:  a. Increase frequency of breastfeeds/ complementary feeds  b. Give animal protein and micronutrient-rich foods (egg, chicken liver, shredded chicken and beef)  c. Add oil to food  d. Increase energy and nutrient density by giving mashed beans instead of the broth and by giving thick papa instead of soup | No intervention | Health facility staff | Health facility | Educaion interventions was delivered for outpatient clinics and children were followed for 180 days | The average age of the children in the intervention and control groups was similar. Despite paired randomization, children from the intervention group had lower family income, social class and  maternal schooling | The nutritional status of the children was very similar between  Groups, except for the mean weight-for-age Z-score  that was significantly higher in the control group, when all  children were considered together | - | 180 d | Intervention grp:  WAZ: -0.18+-0.78  HAZ: -0.37+-0.97    Control: WAZ: -0.25+-0.78  HAZ: -0.41+-0.81 | | |
| **Author** | **Country** | **Region** | **Type of study** | **Age group** | **Intervention- what was the educational message** | **Control** | **Who delivered** | **Where was the education given** | **Duration of intervention** | **Baseline demographics** | **Baseline nutritional status** | **Seasonal variation** | **Duration of follow up** | **Results** | | |
| Roy 2007^30^ | Bangladesh | Rural | cRCT  Effectiveness  Food insecure | children aged 6 to 9 months  who were well nourished or mildly malnourished/  I: 306  C: 305 | Weekly nutrition education based on the nutrition triangle concept of UNICEF for 6 months The messages were prioritized for food security, psychosocial stimulation, and care and health-seeking behavior and were built on the preliminary exploration and focus group. The messages delivered were simple, standardized, and age-appropriate. | regular BINP services | Community health workers/counselors | Community center | Weekly education for 6 months | Similar SES of the two groups at baseline. | At baseline, the mean weight-for-age as a percentage  of the NCHS median was comparable in the intervention and control groups (83.9% vs. 83.6%, respectively; *p* = NS) | - | 6 mo | Intervention grp:  WAZ: − 1.43 ± 0.73; WLZ: − 0.64 ± 0.87  HAZ:   − 1.90 ± 0.93    Control:  WAZ: − 1.90 ± 0.79; WLZ: − 1.14 ± 0.93  HAZ:  − 2.15 ±  0.99 | |  |
