# Supplementary material for: Impact of education and provision of complementary feeding on growth and morbidity in children less than 2 years of age in developing countries: a systematic review
Source: BMC Public Health. 2013 Sep 17;13(Suppl 3):S13. doi: 10.1186/1471-2458-13-S3-S13 (PMC3847349; doi:10.1186/1471-2458-13-S3-S13)
Supplement: Additional File 2 — Characteristics of studies on impact of provision of complementary feeding with or without education. [file 1471-2458-13-S3-S13-S2.docx]

| **Additional file 2: Characteristics of included studies (Complementary feeding with or without education)** | | | | | | | | | | | |  |
| --- | --- | --- | --- | --- | --- | --- | --- | --- | --- | --- | --- | --- |
| **Study ID** | **Country** | **Region** | **Type of study** | **Age group** | **Intervention** | **control** | **Duration of intervention** | **Baseline demographics** | **Baseline nutritional status** | **Seasonal variation** | **Duration of follow up** | **Results** |
| Akalu 2010^32^ | Ethiopia | Rural | cRCT  Effectiveness  (food insecure) | 5-29 mo/  I: 73  C: 78 | **Area A: Wama Banaya**  Quality protein maize (QPM)- maize varieties biofortified with increased lysine and tryptophan levels | Conventional maize | 13 months | The treatment groups did not differ in their socio-demographic  characteristics, except that households in the conventional maize group were more likely to be Protestant (67%) than households in the QPM group  (49%). | - | - | 13 mo  11 months | Intervention grp: WAZ: –0.89 ± 0.87  HAZ: –1.35 ± 1.11  MUAC: –1.35 ± 1.11 Control:  WAZ: –1.15 ± 0.99  HAZ: –1.36 ± 1.25  MUAC: –1.36 ± 1.25 |
|  |  |  |  |  | **Area B: Sibu Siri**  Quality protein maize (QPM)- maize varieties biofortified with increased lysine and tryptophan levels |  | 11 months |  |  |  |  | Intervention grp: WAZ: –0.81 ± 0.91; WHZ: –0.15 ± 1.03 HAZ: –1.26 ± 1.06  MUAC z score: –0.87 ± 0.97  WAZ: –1.01 ± 0.92; WHZ: –0.18 ± 0.91  HAZ: –1.54 ± 1.32  MUAC: –0.91 ± 0.84 |
| Adu- Afarwuah 2007^11^ | Ghana |  | cRCT  Efficacy  Food secure | 5 mo/  I: 97  C: 81 | Nutributter: fortified fat spread modified to include a still larger set of micronutrients plus added energy. (RNI of 14 vitamins and minerals plus some calcium, potassium, phosphorous, magnesium, and manganese as well as energy (108 kcal/ | No intervention | 6 months | The groups did not differ significantly at baseline, except that the NB group had a higher proportion of boys | The children in NB group weighed slightly more than those in the control group. | - | 6 mo | Intervention grp: WAZ -0.40+-1.10  HAZ: -0.14+-1.00 Hb: 114+-14  % Anemia: 10    Control: WAZ: -0.74+-1.10  HAZ -0.40+-1.00  Hb 106+-14  % Anemia 32 |
| Smuts 2005^41^ | South Africa | Rural | RCT  Effectiveness  Food secure | 6 mo/  I: 49  C: 50 | Daily MMN supplements given as foodlet (tablets). Mothers were trained to mix foodlets with a small quantity of porridge (predominantly maize meal), | Placebo | 6 months | The average age of the 194 infants that participated in the study was 8.4 _ 2.0 mo at baseline | There were no significant differences in  nutritional status of the groups at baseline with 40% of infants with anemia (hemoglobin _ 110 g/L), 2% underweight, and  11% stunting | - | 6 mo | Intervention grp: % diarrhea: 22.7  % ARI: 6.8  Hb: 117+-11    Control: % diarrhea 20.9  % ARI 9  Hb 111+-12 |
| Mazariegoes 2004^36^ | Guatemala | Rural | RCT  Effectiveness Food secure | 6-12 mo/  I: 203  C: 207 | The phytate concentration of the test maize was ~80% reduced from the isohybrid control maize. | The phytate content of the control maize was typical of the highphytate  content of this grain, averaging 710 mg/100 g. | 6 months | The intervention arms did not significantly differ at baseline in demographic characteristics. In general, most infants were of low SES and were representative of the general status of the community | - |  | 6mo | I: WAZ -1.30+-1.12  HAZ -2.52+-1.10  C: WAZ -1.30+-1.08  HAZ -2.53+-1.13 |
| Obatolu 2003^37^ | Nigeria | Rural | RCT  Efficacy  Food secure | 4 mo/  I: 30  C: 30 | extruded formulated complementary diet from maize and cowpea (L1A1) | 2 groups of controls: infants of low socioeconomic status without  the feeding intervention (L2N) and infants of above-average  Socio-economic status without the feeding intervention (HN). | 14 months | There were similarities in mothers’ educational level, occupation, and estimated family income between the L1A1 and L2N infants. |  | - | 14 mo | Intervention grp: weight (kg) 10.07+-1.08  Length gain: 79.7+-3.3    Control: weight (kg) 6.84+-1.08  Length gain 73.7+-3.3 |
| Bhandari 2001^33^ | India | Rural | RCT  Efficacy  food secure | 4 mo/ food supplementation grp: 87, nutrition counselling grp: 97,  C: 91 | Children were randomized into 1 of 2 intervention groups:  Nutritional counseling group (NC): 30-45 min monthly counseling with no food supplement  Food supplementation group (FS): received fortified milk-based cereal + nutritional counseling (in addition to usual before and home foods) | **Control #1**: Visitation group (V): home visits 2/wk for morbidity assessment no advice  **Control #2**: Non-intervention group (NI): contacted at 6, 9 and 12 mo for dietary and anthro assessment; no other visits, no advice. | 8 mo | Children from control had almost the same demographics as those in intervention | - | - | 8 mo | I: weight gain 1.93 +-0.57  Height 68.6+-2.9  % Stunted 63.9  C: weight 1.84+-0.72  Height 68.4+-2.4  % Stunted 75.8 |
| Lutter 2006^34^ | South Africa | unclear | RCT  Effectiveness  Food secure | 6-12 mo/  I: 170  C: 149 | Children received enriched bread, powdered skim milk, and vegetable oil as did all other family members. Also received education based on raising awareness about good early child nutrition. | No intervention | 11 mo | - | - | - | 13 mo | I: WAZ -1.00+-0.97  HAZ -1.27+-1.04  % underweight 14.5  % Stunted 23.7  C: WAZ -1.23+-0.94  HAZ -1.42+-1.09  % underweight 24.1  % stunted 27.5 |
| Maluccio & Flores 2004^35^ | Nicaragua |  | cRCT  Effectiveness  Food secure | 0-59 months | Mothers received cash transfers and Education, not specified but based on:  a. Breastfeeding  b. Child feeding  c. Illness care  d. Household sanitation and hygiene; |  | 12 months |  |  |  | 12 months | Mean Hb (g/L) I: 112+-NA  % Anemia 33  C: Mean Hb 114  % Anemia 31 |
| Rivera 2004^39^ | Mexico | Rural | RCT  Effectiveness  Food secure | 0-12 mo/  I: 373  C: 277 | A fortified milk product with recommended daily ration of 44 g (194 kcal energy; 10mg Fe; 10 mg Zn; 400 µg Vit. A), health services and educaton (details not stated) for 2 years. Families received cash transfers for 2 years. | Same as intervention but provided intervention for 1 year, instead of 2 years. | 2 years | There were no significant differences in SES between the two groups. | - | High attrition rate due to unusually long and severe rainy season that resulted in a high rate of temporal emigration from a large number of communities because of damage caused by severe flooding. | 24 months | Mean Hb I: 111  % Anemia 44  C: Mean Hb 108  % Anemia 55 |
| Oelofse 2003^38^ | Zambia | Urban | RCT  Effectiveness  Food secure | 6 mo/  I: 16  C: 14 | Received centrally processed, micronutrient-fortified complementary food (dry cereal and test porridge) 60 g dry product /d equivalent to: 100% RDA vitamin A 80% RDA iron>100% RDA zinc. They received demonstrations on how to prepare the porridge and a measuring spoon to ensure the correct amount of porridge to be consumed. | Continued usual diet. | 6 mo | - | Hb concentrations were similar at baseline between the two groups, but serum iron was slightly higher in the intervention group as compared to control (10.6 vs 9.6) | - | 6 mo | I: WAZ -0.55+-0.99  Height 74.4+-1.8  Mean Hb 108+-9  C: WAZ -0.52+-1.60  Height 74.5+-3.1  Mean Hb 106+-13 |
| Schroeder 2002^40^ | Vietnam | Not clear | RCT  Effectiveness  Food secure | 5-25 mo/  I: 114  C: 118 | Mothers received education on infant feeding, malnourished children received extra food. Details of education were not specified but were based on: Not specified but based on: Community-based  Volunteers Community  a. Breastfeeding centres  b. Food variety  c. Complementary feeding  d. Health care  e. Taking care of healthy children at home | No intervention | 6 mo | Not clear | Not clear | 6 mo |  | I: WAZ -1.92+-0.78  HAZ |
| Zavaleta 2011^42^ | Peru | Peri urban community | RCT  Efficacy  Food secure | 6-11 mo/  I: 253  C: 246 | complementary food (40 g/day) with the protein source being  the milkfat globule membrane (MFGM) protein fraction | complementary food (40 g/day) with the protein source being skim milk proteins | 6 months | the MFGM and control groups also were similar and homogenous with regard to variables of general socioeconomic and demographic status, except for  proportion ofinfants with hemoglobin <105 g/L and potable water facilities; therefore, analyses were controlled for these variables. |  |  |  | % diarrhea- I: 3.84%  C: 4.37%  Hb Mean (sd)  I: 108.1 (8.6)  C: 109.9 (8.3)  Serum ferritin (mean, SD), mg/L:  I: 26.7 (24.0)  C: 26.9 (25.1) |
